# Genome-Wide Association Study of Proneness to Anger

**DOI:** 10.1371/journal.pone.0087257

**Published:** 2014-01-28

**Authors:** Eric Mick, James McGough, Curtis K. Deutsch, Jean A. Frazier, David Kennedy, Robert J. Goldberg

**Affiliations:** 1 Department of Quantitative Health Sciences and the Department of Psychiatry, University of Massachusetts Medical School, Worcester, Massachusetts, United States of America; 2 Division of Child and Adolescent Psychiatry, University of California, Los Angeles Semel Institute for Neuroscience and Human Behavior, David Geffen School of Medicine, Los Angeles California, United States of America; 3 Eunice Kennedy Shriver Center, University of Massachusetts Medical School, Worcester, Massachusetts, United States of America; 4 Psychiatry Department, Division of Child and Adolescent Psychiatry, University of Massachusetts Medical School, Worcester, Massachusetts, United States of America; 5 Psychiatry Department, Division of Neuroinformatics and the Child and Adolescent NeuroDevelopment Initiative, University of Massachusetts Medical School, Worcester, Massachusetts, United States of America; 6 Department of Quantitative Health Sciences, University of Massachusetts Medical School, Worcester, Massachusetts, United States of America; University of Wuerzburg, Germany

## Abstract

**Background:**

Community samples suggest that approximately 1 in 20 children and adults exhibit clinically significant anger, hostility, and aggression. Individuals with dysregulated emotional control have a greater lifetime burden of psychiatric morbidity, severe impairment in role functioning, and premature mortality due to cardiovascular disease.

**Methods:**

With publically available data secured from dbGaP, we conducted a genome-wide association study of proneness to anger using the Spielberger State-Trait Anger Scale in the Atherosclerosis Risk in Communities (ARIC) study (n = 8,747).

**Results:**

Subjects were, on average, 54 (range 45–64) years old at baseline enrollment, 47% (n = 4,117) were male, and all were of European descent by self-report. The mean Angry Temperament and Angry Reaction scores were 5.8±1.8 and 7.6±2.2. We observed a nominally significant finding (p = 2.9E-08, λ = 1.027 - corrected p_gc_ = 2.2E-07, λ = 1.0015) on chromosome 6q21 in the gene coding for the non-receptor protein-tyrosine kinase, Fyn.

**Conclusions:**

Fyn interacts with NDMA receptors and inositol-1,4,5-trisphosphate (IP3)-gated channels to regulate calcium influx and intracellular release in the post-synaptic density. These results suggest that signaling pathways regulating intracellular calcium homeostasis, which are relevant to memory, learning, and neuronal survival, may in part underlie the expression of Angry Temperament.

## Introduction

Anger has been characterized as a complex construct encompassing irritability, hostility, and aggressive behavior. Its components -- impulsive, unprovoked, and developmentally inappropriate outbursts of anger – may be associated with dysregulated emotional control or impaired neural circuits mediating emotion, cognition, and inhibition [1], [2]. Approximately 5% of children and adults in community samples exhibit extreme levels of anger, hostility, and aggression considered to be clinically significant [3]–[5].

Further, these traits are strongly associated with a range of psychiatric symptomatology. Behavioral dysregulation and aggression also predict psychiatric hospitalization and suicidality in childhood and a wide range of psychiatric morbidity in adulthood [6]–[11]. Moreover, dysregulated emotional control and intermittent explosive disorder in adults is associated with a greater lifetime burden of psychiatric morbidity and severe impairment in role functioning [2], [5]. Proneness to anger in the general community is also associated with premature all-cause mortality [12], primarily due to cardiovascular disease [13]–[18]. Consequently, effective primary or secondary interventions that reduce anger, hostility, and aggression might have a substantial public health impact on quality of life and, ultimately, longevity.

Symptoms of deficient emotional self-regulation in adults are familial [19] and, as recently shown in the Vietnam Era Twin Registry, are influenced by moderate genetic effects, particularly on the tendency to switch from euthymia to depression or anger [20]. A commonly used continuously-distributed measure of this trait in children, the Child Behavior Checklist Dysregulation Profile, has been found to be highly heritable with additive genetic effects consistently explaining up to 67% of its variance [3], [4], [21], [22]. A small GWAS of this trait in children with attention-deficit hyperactivity disorder suggested possible associations with several genes implicated in neurodevelopment, synaptic plasticity, as well as hippocampal dependent memory and learning [23]. Taken together, these studies suggest the role of heritable factors in determining individual differences in the self-regulation of emotional and cognitive neural circuits, ones that may underlie proneness to anger, hostility, and aggression. The primary goal of this study was to identify genetic susceptibility loci for proneness to anger through a secondary analysis of publically-available data.

## Materials and Methods

Phenotypes and genotypes were downloaded from the National Center for Biotechnology Information (NCBI) database of genotypes and phenotypes (dbGaP) [24] for the NHLBI funded Atherosclerosis Risk in Communities (ARIC) Study (Accessions: phs000280.v1.p1 and phs000090.v1.p1). In accord with the restrictions on the use of the data defined by participant informed consent agreements, this ARIC dataset may be used for general research use following the approval of the ARIC Data Access Committee and IRB approval at the approved investigators institution. The acquisition and use of these data was approved and overseen by the Human Subjects Institutional Review Board at the University of Massachusetts Medical School.

### Atherosclerosis Risk in Communities (ARIC) Cohort

Details regarding the design and objectives of the Atherosclerosis Risk in Communities Study have been previously published and are available online (http://www.cscc.unc.edu/ARIC/)[25]. In brief, this is a large population-based sample of 15,792 individuals ascertained from a probability sample of four U.S. communities between 1987 and 1989. Subjects were followed every three years for several re-assessments (1990–1992, 1993–1995, and 1996–1998) to study the incidence and course of atherosclerosis in men and women aged 45–64 years at the time of initial clinic assessment.

### Genotyping

Samples were genotyped on the Affymetrix Genome-Wide Human SNP Array 6.0 at the Broad Institute Center for Genotyping and Analysis for the Gene Environment Association Studies initiative (GENEVA, http://www.genevastudy.org). Genotypes of 12,771 ARIC participants were submitted to dbGaP and were available for analysis. For the present analyses, we first excluded related individuals (n = 927; 11,844 remaining); subsequently, subjects with missing data on the Spielberger Trait Anger Scale (n = 599; 11,245 remaining). Twenty percent of ARIC participants were African American (AA) by self-report and the remaining subjects classified only as “White” (i.e., individuals of European ancestry (EA)). Using the online genetic power calculator toolset [26], we determined the statistical power to detect association at a quantitative trait locus (QTL) to account for at least 1% of variance in the EA subsample to be 0.81; however, the power to detect genome-wide statistically significant associations in the AA subgroup was substantially lower, at 0.21. Therefore, we focused exclusively on the EA sample (with n = 2,498 excluded and n = 8,747 remaining).

### Quality Control

Extensive quality control checks were conducted on the original data and we downloaded the cleaned dataset with genotypes flagged for chromosomal abnormalities (n = 840,606 SNPs). Within the selected sample of 8,747 subjects, we filtered SNPs by minor allele frequency (MAF) conditional on call rate (CR) including SNPs with: 0.01 ≤ MAF < 0.05 and CR >99%; 0.05 ≤ MAF <0.1 and CR >97%, MAF ≥0.1 and CR >95% (n = 148,142 SNPs excluded). Any SNPs found to be out of Hardy-Weinberg Equilibrium (p<1.0E-4) were excluded from further consideration (n =  14,821 SNPs excluded). After applying the described quality control filters, the final sample consisted of 677,643 SNPs in 8,747 unrelated subjects.

### Spielberger State-Trait Anger Scale

The Spielberger State-Trait Anger Scale is a Likert-type four-level self-rating scale (1 = almost never, 2 = sometimes, 3 = often, 4 = almost always) [27]. Factor analysis of the Trait Anger items have yielded two weakly correlated (r^2^ = 0.29) factors labeled “Angry Temperament” and “Angry Reaction” [28]. The former refers to the propensity of individuals to express anger frequently, with little or no provocation, and includes four items: 1) I am quick tempered, 2) I have a fiery temper, 3) I am a hotheaded person, and 4) I fly off the handle. In contrast, the Angry Reaction subscale reflects frustration in response to criticism or mistreatment; it also includes four items: 1) I get angry when I am slowed down by others’ mistakes, 2) I feel annoyed when I am not given recognition for doing good work, 3) It makes me furious when I am criticized in front of others, and 4) I feel infuriated when I do a good job and get a poor evaluation. The items for each subscale are summed to generate specific scores each ranging from 4 to 16. Temperament scores above 8, and Reaction scores above 10, are considered to be elevated and have been assessed as risk factors for coronary heart disease outcomes in prior analyses of these data [14].

The Spielberger State-Trait Anger scale was first adminstered at visit 2 (1990–1992) in 8,747 subjects and again at visit 3 (1993–1995) in 7,246 subjects; moderate to strong test-retest reliability for the Angry Reaction (ICC = 0.50) and the Angry Temperament (ICC = 0.65) subscales was observed. Subjects with missing Spielberger State-Trait Anger scale data at visit 3 (n = 1,501) had slightly higher visit 2 scores on the Angry Temperament (6.0±1.9 vs. 5.7±1.7, p<0.0001) but not on the Angry Reaction (7.6±2.2 vs. 7.6±2.1, p = 0.7) subscales. To maximize the sample size, and to avoid misclassification of Angry Temperament at visit 3, we utilized information collected at visit 2 in this report.

### Statistical Analysis

All genome-wide association analyses were conducted using PLINK [29] employing datasets downloaded and filtered as described earlier under genotyping. To control for multiplicity in the number of SNPs tested, we adopted the conservative recommendation of Dudbridge et al [30] and Pe’er et al [31], considering p-values less than 7.0E-08 to be statistically significant genome-wide. Our primary test of association was for the additive effects of minor allele dosage on the quantitative summary scores of Angry Temperament and Angry Reaction (each ranging from 4-16 points) in the selected sample. To further estimate magnitude and direction of effect, we then conducted case-control analyses (i.e., Angry Temperament >8 vs. ≤8 or Angry Reaction >10 vs. ≤10) for SNPs of interest from the quantitative association results. The potential for inflation of the test statistic due to population heterogeneity was estimated with the lambda statistic (defined as the observed median statistical test divided by the expected median statistical test) and with quantile-quantile plots of observed and expected p-values. To adjust for population stratification, we conducted genome-wide association tests on the model residuals generated for each anger phenotype regressed on the principal components representing genetic substructure provided by the ARIC investigators through dbGaP.

### Gene Ontology (GO) Enrichment Analyses

Genetic enrichment or pathway analysis was conducted with the *INRICH* pathway analysis tool for GWAS, designed for detection of enriched association signals of linkage-disequilibrium (LD) independent genomic regions within biologically relevant gene sets (http://atgu.mgh.harvard.edu/inrich) [32]. Independent LD association intervals were identified by the “clumping” algorithm in PLINK [29]. This algorithm identifies genomic regions surrounding index SNPs (i.e. p<5E-05) defined by nearby SNPs (within 250 kb) that are in linkage disequilibrium (r^2^>0.4) with the index SNP and are also nominally statistically significant (p<5E-03). These association regions were referenced against genetic categories defined by the Gene Ontology project (http://www.geneontology.org/) according to known biological processes, cellular components, and molecular function of their gene products. For each GO term (e.g., “regulation of synaptic plasticity”) the number of association intervals containing genes associated with that GO term were counted to determine if the proportion of overlapping intervals is greater than expected by chance through multiple permutation (n = 5,000 permutations). The permutation procedure places each association interval at random genomic locations but conditions on SNP/gene density to control for potential bias associated with gene size and SNP density.

## Results

Data from 8,747 Caucasian men and women participating in the ARIC study were included in our genome-wide association analyses. Subjects were, on average 54.3±5.7 (range 45–64 years) years old at baseline enrollment, 47% (n = 4,117) were men, and all were of European descent by self-report. The mean Angry Temperament and Angry Reaction scores were 5.8±1.8) and 7.6±2.2, respectively. Elevation of the Angry Reaction subscale (>10, n = 845, 9.7%) was seen more often than elevation of the Angry Temperament subscale (>8, n = 515, 5.9%).

**Figure 1:**
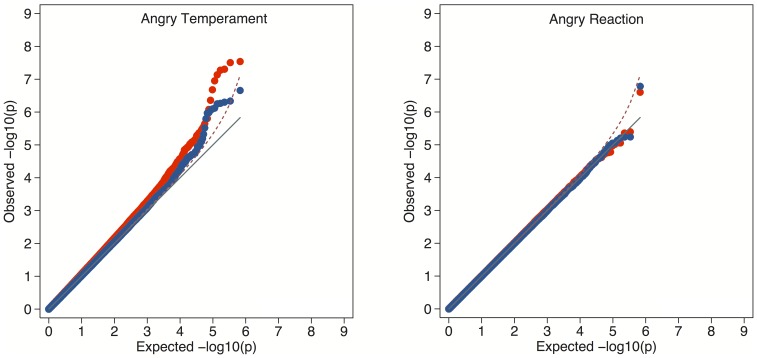
Quantile-Quantile (QQ) plot of the genome-wide association results for the Spielberger State-Trait Anger Scale subscales. The red symbols depict results from analysis uncorrected for ancestry, the blue symbols depict results corrected for ancestry (i.e. P_GC_), and the dashed red line depicts the 95% confidence interval for the distribution of results. There was no evidence of inflation of the test statistic for the Angry Reaction scores in the uncorrected (λ  = 0.9998) or the corrected (λ  = 1.0029) analyses. For the Angry Temperament score test statistics, there was slight inflation of the uncorrected analysis (λ  = 1.0272) that was further reduced with correction for genetic ancestry (λ  = 1.0015).

Association with each anger phenotype was assessed across the 677,643 SNPs filtered by call rate conditional on minor allele frequency and deviation from Hardy-Weinberg equilibrium. We observed a potentially statistically significant finding (smallest p = 2.9E-08) for Angry Temperament but not for Angry Reaction (smallest p = 2.5E-07). The QQ plot (Figure 1) shows the distribution of expected p-values against the observed distribution for both Angry Temperament and Angry Reaction. Inspection of the Angry Temperament plot clearly reveals a greater number of significant findings than expected by chance with the distribution of p-values being only slightly inflated (lambda = 1.027). Adjustment for genetic background reduced lambda to 1.0015 and attenuated the uppermost findings (smallest p_GC_ = 2.2E-07) but the departure from the diagonal in the tail of both the corrected and uncorrected QQ plots indicates enrichment of significant associations (Figure 1).

Table 1 lists the top 38 association regions defined by an index SNP with nominal p<5E-05 and at least one additional proximal SNP (<250 kb) in LD (r^2^>0.4) and significant at p<5E-03 for the Angry Temperament subscale. We assessed enrichment of Gene Ontology (GO) terms overlapping with association regions using the INRICH algorithm to control for potential biases caused by SNP/gene density and gene size. There was evidence of enrichment for no GO targets among the top regional associations for Angry Reaction scores (Table 2), but we found some nominal evidence of enrichment for 6 GO targets (Table 3) in the Angry Temperament association regions in Table 1 (none survived correction for the number of targets tested). Among the targets nominally significant at p<0.05, four genes overlap and are present in more than one target: *ABAT* (4-aminobutyrate aminotransferase), *FYN* (Fyn oncogene related to Src, Fgr, Yes), *PDE3A* (phosphodiesterase 3A, cGMP-inhibited), and *VEGFC* (vascular endothelial growth factor C).

**Table 1:** Genome-Wide Association Results for the Angry Temperament subscale.

					Temperament Score			Case-Control		LD-Based Association Interval		
CHR	SNP	A1	BP	MAF	Beta	P-RAW	P-GC*	OR	P	SNPs	Length kb	Genes
6	rs2148710	T	112,228,920	0.137	0.22	2.9E-08	4.6E-07	1.26	0.0075	12	135.32	FYN
7	rs6954895	G	35,552,732	0.257	0.16	2.1E-07	4.7E-06	1.24	0.0029	7	23.96	-
6	rs2844775	A	30,287,400	0.250	–0.15	8.3E-07	7.9E-05	0.87	0.0617	31	335.03	PPP1R11,RNF39,ZNRD1,TRIM40,TRIM31,TRIM26, TRIM15,TRIM10,HLA-A29.1,HCG9
6	rs670292	C	150,744,544	0.466	0.13	1.6E-06	2.2E-07	1.24	0.0009	7	31.56	IYD
11	rs16924133	A	33,281,952	0.028	0.38	1.7E-06	2.1E-05	1.79	0.0001	4	245.99	TCP11L1,HIPK3,CSTF3
10	rs12249434	A	118,288,696	0.088	0.22	4.3E-06	1.2E-05	1.39	0.0013	3	50.64	PNLIP,PNLIPRP1
4	rs6834498	A	189,802,160	0.181	–0.16	4.9E-06	1.6E-05	0.86	0.0808	9	20.41	-
20	rs6012564	G	46,975,008	0.406	0.12	6.7E-06	1.1E-05	1.25	0.0007	12	242.31	STAU1,CSE1L,ARFGEF2
16	rs1299926	T	8,778,582	0.054	0.26	7.8E-06	2.3E-05	1.54	0.0003	2	5.12	ABAT
19	rs8102754	A	34,180,880	0.454	0.12	8.3E-06	8.9E-05	1.24	0.0010	2	7.32	-
1	rs16840114	C	238,692,048	0.060	0.25	9.1E-06	6.7E-05	1.39	0.0055	2	0.55	FMN2
1	rs4628571	C	208,925,024	0.449	–0.12	1.0E-05	9.3E-05	0.83	0.0048	11	57.03	KCNH1,HHAT
20	rs238215	T	47,303,912	0.235	–0.14	1.2E-05	6.4E-06	0.79	0.0036	9	120.68	ZNFX1,STAU1,DDX27
4	rs11724215	G	89,020,656	0.070	0.23	1.3E-05	2.1E-04	1.42	0.0019	4	40.93	MEPE
6	rs782000	G	71,408,536	0.272	0.13	1.4E-05	6.0E-05	1.27	0.0006	3	149.06	SMAP1
4	rs17084746	C	52,666,560	0.011	0.55	1.8E-05	8.2E-05	1.82	0.0125	4	83.61	SPATA18
6	rs7742473	A	119,208,432	0.098	0.19	1.9E-05	2.3E-05	1.21	0.0610	5	13.40	-
20	rs1883881	T	46,999,184	0.349	–0.12	2.0E-05	1.6E-05	0.78	0.0004	12	245.59	STAU1,CSE1L, ARFGEF2
12	rs10770687	G	20,701,778	0.348	–0.12	2.3E-05	2.9E-04	0.92	0.2281	7	48.40	SLCO1C1,PDE3A
11	rs4758435	C	5,798,432	0.190	0.14	2.5E-05	9.8E-05	1.25	0.0041	2	1.28	OR52N2
1	rs2495053	C	13,912,617	0.053	0.25	2.6E-05	1.1E-04	1.35	0.0181	3	134.60	PRDM2
1	rs6668091	T	239,578,176	0.143	0.16	2.6E-05	1.7E-04	1.22	0.0221	5	33.04	RGS7
4	rs41501449	C	178,024,608	0.105	–0.18	2.7E-05	1.8E-04	0.71	0.0033	14	173.27	VEGFC
14	rs17831706	T	51,825,136	0.090	0.20	2.7E-05	3.7E-05	1.43	0.0003	3	34.52	PTGDR
1	rs10912593	A	169,454,688	0.315	0.12	3.0E-05	4.8E-05	1.20	0.0062	3	39.34	FMO2
1	rs3889128	G	53,781,820	0.235	0.13	3.1E-05	3.7E-05	1.17	0.0319	3	0.14	GLIS1
2	rs2341997	G	16,835,468	0.440	0.11	3.3E-05	1.5E-04	1.07	0.3060	2	7.48	-
9	rs7035071	C	2,069,250	0.200	–0.14	3.5E-05	2.1E-04	0.85	0.0461	2	7.86	SMARCA2
7	rs10258797	A	121,796,040	0.207	0.14	3.6E-05	3.1E-04	1.29	0.0008	4	46.00	CADPS2
12	rs215996	C	2,589,558	0.182	0.14	3.6E-05	1.9E-04	1.20	0.0203	5	17.18	CACNA1C
6	rs583807	T	4,100,791	0.081	–0.20	3.7E-05	2.0E-05	0.71	0.0094	5	65.89	-
1	rs17373189	C	172,636,384	0.157	–0.15	3.9E-05	1.7E-04	0.75	0.0026	17	358.45	RABGAP1L,GPR52
18	rs17596183	A	34,730,256	0.363	–0.11	4.1E-05	1.7E-04	0.86	0.0294	10	31.34	-
23	rs2354304	A	94,283,280	0.432	0.09	4.2E-05	7.1E-05	1.29	0.0008	12	23.20	-
23	rs2272657	A	48,203,844	0.374	0.09	4.5E-05	4.0E-04	1.22	0.0103	3	10.48	SLC38A5
23	rs543042	A	146,406,080	0.142	–0.13	4.5E-05	1.5E-05	0.72	0.0061	17	143.24	-
8	rs7843469	A	69,360,592	0.440	0.11	4.6E-05	1.5E-04	1.14	0.0420	6	23.21	-
11	rs2712799	G	74,526,864	0.379	0.11	4.8E-05	1.0E-04	1.10	0.1340	14	74.05	SLCO2B1,OR2AT4

* p-values results from phenotypes adjusted for principal components representing genetic substructure of ARIC participants.

**Table 2:** Genome-Wide Association Results for the Angry Reaction subscale.

						Reaction Score			Case-Control		LD-Based Association Interval		
CHR	SNP	A1	BP	MAF	P-HWE	Beta	P-RAW	P-GC*	OR	P	SNPs	Length (kb)	Interval Genes
23	rs3752433	A	22,149,640	0.312	0.392	0.15	2.5E-07	1.6E-07	1.181	0.0067	14	41.451	PHEX
6	rs555017	T	20,293,030	0.369	0.065	0.15	4.0E-06	8.8E-06	1.221	0.00012	3	8.696	MBOAT1
2	rs7578047	C	68,433,432	0.142	0.670	–0.22	4.4E-06	5.8E-06	0.7164	0.000043	7	62.651	PLEK
4	rs2045797	C	103,572,096	0.422	0.915	–0.15	1.1E-05	1.0E-05	0.8443	0.0012	16	109.868	SLC39A8
3	rs17535407	C	105,381,760	0.406	0.003	0.15	1.8E-05	1.0E-05	1.176	0.0018	2	8.094	-
16	rs3922878	C	85,412,208	0.034	0.006	–0.38	2.5E-05	3.5E-05	0.5534	0.00091	3	12.691	-
8	rs3110145	T	60,204,964	0.138	0.013	–0.20	2.5E-05	2.4E-05	0.7081	0.00003	9	129.854	TOX
16	rs10863202	A	84,545,496	0.301	0.139	0.15	2.7E-05	6.0E-05	1.165	0.0053	7	6.522	-
10	rs11201163	A	86,420,712	0.330	0.815	–0.14	2.7E-05	2.8E-05	0.8214	0.00044	5	36.769	-
3	rs12639503	T	51,835,856	0.023	0.148	0.45	3.2E-05	2.6E-05	1.522	0.0045	4	71.18	IQCF2,IQCF3
7	rs10271531	T	80,951,880	0.057	0.501	0.29	3.6E-05	3.5E-05	1.485	0.000037	9	160.549	-
8	rs2572430	T	11,142,714	0.450	0.071	–0.14	3.7E-05	3.9E-03	0.8764	0.011	13	78.835	MTMR9
3	rs1387024	C	114,030,288	0.351	0.699	–0.14	3.7E-05	4.1E-05	0.8734	0.013	6	141.936	CD200R1,CD200R2
8	rs7012323	G	127,651,008	0.247	0.867	–0.16	3.9E-05	3.6E-05	0.8366	0.0037	2	3.323	-
8	rs7820917	T	9,685,278	0.378	0.402	–0.14	3.9E-05	2.9E-03	0.9107	0.078	18	378.211	TNKS
3	rs1878012	C	21,822,622	0.213	0.536	0.16	4.4E-05	3.8E-05	1.251	0.00016	3	4.205	-
9	rs1160245	A	119,032,744	0.490	0.025	0.13	4.4E-05	5.9E-05	1.197	0.00046	17	38.647	ASTN2
5	rs6874556	C	132,619,432	0.426	0.330	0.13	5.0E-05	4.7E-05	1.101	0.061	4	17.986	FSTL4

* p-values results from phenotypes adjusted for principal components representing genetic substructure of ARIC participants.

**Table 3:** Gene Ontology (GO) Enrichment Results.

GO Target Category	Empirical p-value	Corrected p-value	Associated Genes
GO:0045776 Negative regulation of blood pressure	.00039	0.30	ABAT, VEGFC
GO:0043252 Sodium-independent organic anion transport	.00059	0.35	SLCO2B1, SLCO1C1
GO:0004386 Helicase activity	.0088	0.88	SMARCA2, DDX27
GO:0045471 Response to ethanol	.017	0.95	ABAT, FYN
GO:0042493 Response to drug	.029	0.98	ABAT, VEGFC, PDE3A
GO:0030168 Platelet activation	.030	0.98	FYN, VEGFC, PDE3A

## Discussion

In this study we conducted a genome-wide association study of trait anger in a large sample of middle-aged and elderly men and women recruited from 4 large U.S. communities. We found evidence of genetic susceptibility for the anger trait associated with a proclivity for unprovoked (i.e., Angry Temperament scores) but not provoked (i.e., Angry Reaction scores) anger. In contrast to the Angry Reaction results, for Angry Temperament we identified a greater than expected number of extreme p-values and nominal evidence of GO target enrichment.

We have previously studied mood dysregulation using data from the ADHD sub study of the Psychiatric Genomics Consortium (PGC) [23]. In this study of 341 referred ADHD assessed with the Child-Behavior Checklist [23], [33], we found suggestive but weak associations with *BDNF* (brain-derived neurotrophic factor), it’s preferred receptor (*NTKR2*, neurotrophic tyrosine kinase receptor, type 2), and a scaffolding protein (*LRRC7,* leucine rich repeat containing 7) anchoring a downstream protein kinase (*CAMK2A*, calcium/calmodulin-dependent protein kinase II alpha) required for initiation and maintenance of early-long-term potentiation [34], [35]. We also found preliminary evidence of association with prion protein (*PRNP*) and it’s ligand (*STIP1;* stress-induced-phosphoprotein) that together mediate astrocyte differentiation/survival [36], [37] and homeostatic function of hippocampal circuits [38]. Disruption of STIP1-PRPN or BDNF-NTKR2 binding in the hippocampus [39]–[41] impair long-term potentiation, spatial learning, memory consolidation, and hippocampal development.

The most statistically significant association in the current study was with rs2148710 and the Angry Temperament score in *FYN.* Interestingly, the mechanisms underlying the pathways suggested by our small studies of mood dysregulation in children are dependent on Fyn activity. For example, post-synaptic BDNF-NTRK2 binding activates Fyn to phosphorylate NDMA-receptors (N-methyl-d-aspartate) and increases subsequent calcium influx associated with long-term potentiation initiation [35], [42], [43]. Fyn also phosphorylates endoplasmic reticulum inositol-1,4,5-trisphosphate (IP3)-gated channels to stimulate the release of intracellular calcium [44], [45] in response to both NTKR2 [35] and PRNP [46] activity.

Loss of Fyn function in mice has also been associated with blunted long-term potentiation at hippocampal synapses and impaired learning and memory on the hidden platform water maze task [47]. These findings are consistent with learning-disordered/transactional model of explosive anger in which lagging higher-order cognitive skills play a central role [48], [49]. Specific executive functioning deficits could contribute to explosive reactivity through inefficient encoding of previous consequences of noncompliance, thereby interfering with the ability to anticipate consequences of potential actions [49].

The Psychiatric Genomics Consortium (PGC) [50] has recently published cross- disorder analyses documenting pleiotropic effects of associated genes for psychiatric disorders that suggest a common genetic susceptibilities that underlie psychiatric morbidity. These pleiotropic genetic variants in the PGC analyses were enriched for brain expression quantitative trait loci (eQTL) and more specifically for calcium channel activity genes facilitating transmembrane ion diffusion (GO:0005262, calcium channel activity). Interestingly, we also observed a nominal association with a SNP in *CACNA1C* and Angry Temperament in our association analyses (Table 1). *CACNA1C* encodes the **α** subunit of the L-type voltage-gated calcium channel and is a strong candidate for both bipolar disorder and general psychiatric morbidity [32], [50]. Thus, our results may suggest that regulation of calcium-dependent intracellular signaling could play a role not only for psychiatric morbidity but also for variable expression of normative symptoms such as anger.

GO enrichment analyses suggest that there may be shared genetic susceptibility for affect regulation and cardiovascular disease, as well. Using these data, other groups have documented an increased risk for coronary heart disease with an elevated proneness to anger [13], [14], [17], [51], [52], and we have found nominally significant (p<0.05) enrichment of genes associated with the negative regulation of blood pressure (GO:0045776; *ABAT* and *VEGFC*) and platelet activation (GO:0030168; *FYN*, *VEGFC*, and *PDE3A*) in our study of Angry Temperament (Table 3). In addition to impacting hippocampal memory and learning, Fyn is involved with the regulation of platelet shape/response [53] and cardiac myocyte excitability by modulating voltage-gated cardiac sodium channels [54]. Similarly, ABAT, a metabolizer of the inhibitory neurotransmitter GABA, is associated with negative regulation of blood pressure and has previously been associated with disorders (e.g., schizophrenia [55] and autism [56]) that are associated with angry, aggressive behavior. Though the overlapping patterns of association might represent pleiotropic genes, they also might be the result of a confounding by phenotypic correlation (e.g., between affect regulation and cardiovascular disease) [57].

We have evaluated these results in the context of methodological limitations, including considerations that (a) replication studies for this phenotype are not yet available, (b) the measures demonstrated moderate test-retest reliability, and (c) our available sample size may be inadequate to identify genome-wide statistically significant associations that survive correction for population stratification. This may weaken the strength of association for the regions identified and, consequently, no targets survived correction for the number of GO categories evaluated for enrichment. (4) Another limitation exists with respect to the range of ethnicity: there was only a modest number of African American subjects, thus we limited our analyses and generalizability to individuals of European descent.

These limitations notwithstanding, the differences in the distribution of results for the Angry Temperament and Angry Reaction phenotypes are noteworthy. The null results observed for Angry Reaction scores (i.e., an absence of any inflation of test statistic, extreme p-values, or evidence of GO enrichment among top findings) suggest that the associations observed for Angry Temperament may not be due to chance alone, particularly since both scores were generated from a single questionnaire. Compared to those of the Angry Reaction phenotype, the behavioral characteristics measured by the Angry Temperament items (unprovoked, frequent, and extreme anger) more closely resemble the behavioral characteristics of the childhood phenotype --- ones that resulted in putative associations with genes involved in hippocampal synaptic plasticity, memory and learning [23]. The substantial clinical impact of this form of dysregulated emotion on interpersonal functioning [5], [11] and cardiovascular health [14], [15] strongly suggests that additional etiologic research is indicated in order to identify targets for primary and secondary interventions across the life-cycle.

## Acknowledgments

Phenotypes and genotypes were downloaded from the National Center for Biotechnology Information (NCBI) database of genotypes and phenotypes (dbGaP) for the NHLBI funded Atherosclerosis Risk in Communities (ARIC) Study (Accessions: phs000280.v1.p1 and phs000090.v1.p1). The authors thank the staff and participants of the ARIC study for their important contributions.
